# A Thermostabilized, One-Step PCR Assay for Simultaneous Detection of* Klebsiella pneumoniae* and* Haemophilus influenzae*

**DOI:** 10.1155/2017/7210849

**Published:** 2017-03-12

**Authors:** Nur Amalina Khazani, Nik Zuraina Nik Mohd Noor, Chan Yean Yean, Habsah Hasan, Siti Suraiya, Suharni Mohamad

**Affiliations:** ^1^Department of Medical Microbiology and Parasitology, School of Medical Sciences, Universiti Sains Malaysia, Kota Bharu, Malaysia; ^2^School of Dental Sciences, Universiti Sains Malaysia, Kota Bharu, Kelantan, Malaysia

## Abstract

*Klebsiella pneumoniae *and* Haemophilus influenzae *are two common pathogens associated with respiratory tract infections. The identification of these pathogens using conventional molecular diagnostic tests requires trained personnel, cold-chain transportation, and storage-dependance, which does not render them user-friendly. The aim of this study was to develop a thermostabilized, cold-chain-free, one-step multiplex PCR for simultaneous detection of* K. pneumoniae *and* H. influenzae. *The multiplex PCR assay was designed to amplify the* php *gene of* K. pneumoniae *(202 bp) and* p6 *gene of* H. influenzae *(582 bp). In addition, the specific primer to amplify* glm *gene of* Helicobacter pylori *(105 bp) was included as an internal amplification control. Subsequently, the designed primers and all PCR reagents were thermostabilized by lyophilization. The stability of the thermostabilized PCR was evaluated using the* Q*^10^ method. The sensitivity and specificity of performances for thermostabilized PCR were evaluated using 127 clinical isolates and were found to be 100% sensitive and specific. The thermostabilized PCR mix was found to be stable for 30 days and the* Q*10 accelerated stability was found to be 3.02 months. A cold-chain-free, PCR assay for easy, rapid, and simultaneous detection of* K. pneumoniae *and* H. influenzae *was successfully developed in this study.

## 1. Introduction

*Klebsiella pneumoniae *and* Haemophilus influenzae *are two common pathogens associated with respiratory tract infections. Rapid detection and identification of these two pathogens are important so that appropriate and early antimicrobial therapy can be provided. The definitive identification of bacterial respiratory pathogens is made through the culture method. However, the major drawbacks of conventional culture methods are that it is time consuming and laborious and may take at least 48 hours for organism identification. Furthermore, the culture method is insensitive and has a lower positivity rate due to various reasons including the administration of antimicrobial therapy before clinical sample collection and also the low count of causative agents itself [1]. To make things worse, some of the causative agents are very fastidious and require special transport and culture media to grow [2].

Molecular approaches such as polymerase chain reaction (PCR) based methods have become an important tool to detect respiratory pathogens due to their superior sensitivity, relatively faster turnaround time as compared to conventional method, and the ability to identify pathogens that are growing slowly or are difficult to culture. Both conventional and real-time PCR assays have been developed to detect respiratory pathogens [3–9]. However, real-time PCR does not appear to offer advantages when testing a large number of samples due to higher cost of equipment and reagents and the need of trained technical staff and controlled environment for assay setups. In low resource laboratories, conventional PCR have been widely used to detect such pathogens. However, some of the limitations of conventional PCR are the risk of contamination due to multiple pipetting steps, requiring skilled personnel for master mix preparation, and cold-chain transportation and storage. Thus, the clinical application of these techniques in low resources and remote areas is limited due to the need for well-trained personnel and the lack of good facilities to store all required reagents and enzyme in a specific temperature range [3].

In order to overcome the above-mentioned problems, a thermostabilized, ready-to-use one-step PCR assay, which require no cold-chain, was developed in this study for simultaneous detection of* K. pneumoniae *and* H. influenzae *in a single tube reaction. This technique only requires two pipetting steps and thus may reduce the chance of cross-contamination caused by multiple pipetting steps.

## 2. Materials and Methods

### 2.1. Clinical Samples and Clinical Isolates

Two reference bacterial strains used in this study were* Haemophilus influenza* American Type Control Centre (ATCC 49247) and* Klebsiella pneumoniae* (ATCC 1706). For the assessment of sensitivity and specificity, a total of 127 clinical isolates from various bacterial strains were obtained from the Department of Medical Microbiology and Parasitology, School of Medical Sciences, Universiti Sains Malaysia (Table 1), from January to July 2015. All those clinical isolates were identified by VITEK 2 system (BioMérieux, France). The study was approved by the Institutional Review Board of the Human Research Ethics Committee, USM (HREC) IRB Reg No 00004494.

### 2.2. Primer Design

Two specific sets of primers were designed based on the* p6 *gene of* H. influenzae *(582 bp) and* php *gene of* K. pneumoniae *(202 bp) by using the Primer-BLAST program (https://www.ncbi.nlm.nih.gov/tools/primer-blast/). An internal control (IC) primer was designed based on the* glmM *gene of* Helicobacter pylori *(105 bp). This gene is nonvirulent and genetically unrelated to both* K. pneumoniae *and* H. influenzae. *The IC was incorporated in order to verify the absence of PCR inhibitors that could lead to false-negative results. The primers were synthesized by First Base Laboratories, Singapore. Due to issues related to intellectual property and patent, the full sequence of the reverse and forward primers could not be revealed here.

### 2.3. DNA Extraction

The DNA was extracted from reference bacterial strains and clinical isolates using a QIAamp DNA Mini Kit (Qiagen, Germany), according to the manufacturer's instruction. The DNA concentration and purity were measured using a spectrophotometer (Eppendorf Biophotometer, Germany), while the integrity of the extracted DNA was tested using a FluoroSafe-stained agarose gel. Purified DNA was kept at −20°C until it was used.

### 2.4. Thermostabilization of PCR Reagents

Each PCR master mix consisted of 1.25 pmol of each forward and reverse primer for* php *and* p6 *genes, 2.5 pmol of each primer for IC, 1x* Taq *Buffer (Axon Scientific, Malaysia), 1.5 mM MgCl_2_ (Axon Scientific, Malaysia), 0.16 mM of each of the dNTPs (Axon Scientific, Malaysia), and 1.5 U of* Taq* DNA Polymerase (Axon Scientific, Malaysia) and 1 pg IC DNA template.

The positive control contained both DNA templates of* K. pneumoniae *and* H. influenzae *that were added to the mixture; meanwhile the negative control contained only IC. Trehalose (Sigma-Aldrich, USA) was added as an enzyme stabilizer in the PCR mixture and was subjected to a drying process for 2 hours at 5.0^−2^ mBar pressure using Heto vacuum concentrator (Heto Holten A/S, Denmark), which was connected to a LyoLab 3000 freeze-dryer (Heto Holten A/S, Denmark). Then, the thermostabilized PCR tubes were stored in a sealed aluminium pouch with desiccant at 4°C, 25°C, and 37°C.

### 2.5. Optimization of Thermostabilized Multiplex Mixture (Enzyme Stabilizer and* Taq* DNA Polymerase)

Optimization of thermostabilized multiplex PCR was carried out by preparing the standardized multiplex mixture. The enzyme stabilizer was added to thermostabilized PCR mixture in order to preserve the* Taq *DNA polymerase enzyme activity. In this study, the concentration of enzyme stabilizer was optimized starting from 2 to 10%. The concentration of* Taq *DNA polymerase should be in balance with the enzyme stabilizer to make sure it works optimum and does not influence the efficacy and sensitivity of the amplification process. The concentration of* Taq *DNA polymerase was optimized starting from 100 to 250% of* Taq *DNA polymerase. DNA templates of* H. influenzae *and* K. pneumoniae *reference strains were used as positive control.

### 2.6. Thermostabilized Multiplex PCR

The thermostabilized multiplex PCR was performed by adding 18 *μ*l DNase-free water and 2 *μ*l of the DNA template into a tube containing thermostabilized PCR reagents. The mixture was vortexed briefly to dissolve the dried pellets. Each of the negative control tube received 20 *μ*l DNase-free water. The PCR amplification was done using a Gradient Cycler MJ Research (MJ Research, USA) with one cycle of initial denaturation at 95°C for 5 min, 30 cycles of denaturation, and annealing and extension at 95°C for 30 s, 60°C for 30 s, and 72°C for 30 s, respectively. This was followed by an extra annealing temperature at 60°C for 30 s and final extension at 72°C for 3 min. The amplified PCR products were analyzed using 1.5% agarose gel electrophoresis, stained with FluoroSafe (First Base, Singapore), and visualized under UV illuminator using an image analyzer (ChemilImager 5500; Alpha Innotech, San Leandro, CA, USA).

### 2.7. Evaluation of Sensitivity and Specificity of Thermostabilized PCR

Purified DNA of reference strains of* H. influenzae *and* K. pneumoniae *were diluted to an undetectable level for sensitivity determination. The sensitivity of thermostabilized PCR was determined using extracted DNA from 20 clinical isolates of each of* H. influenzae *and* K. pneumoniae*. Limit detection (LOD) of thermostabilized multiplex PCR at the bacterial level was determined using tenfold dilution starting from 10^5^ CFU/ml to 10^1^ CFU/ml. Bacteria cells of both bacteria were grown in BHI broth, extracted by boiling method at 100°C, and used as a template in the assay. The specificity of thermostabilized PCR was also evaluated using extracted DNA from 47 clinical isolates of Gram-negative bacteria and 40 clinical isolates of Gram positive bacteria that are normally found in the respiratory tract.

### 2.8. Evaluation of Stability of Thermostabilized PCR

The stability or shelf life of the thermostabilized PCR assay was estimated using the* Q*10 accelerated aging technique, as described by Clark, 1991 [9]. The thermostabilized PCR tubes were stored under elevated temperatures of 4°C, 25°C, and 37°C up to 30 days and were tested periodically. The duration of thermostabilized PCR was then calculated to estimate its stability at 37°C.

## 3. Results

### 3.1. Multiplex PCR

**Figure 1:**
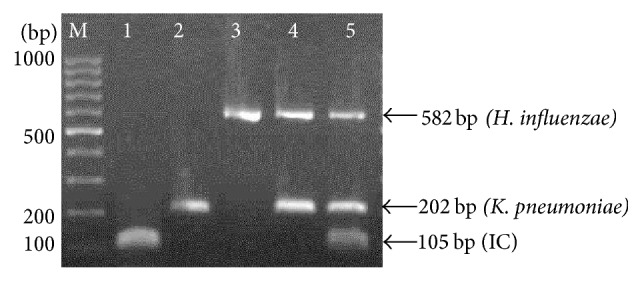
Representative agarose gel electrophoresis of the multiplex PCR using reference strains of* H. influenzae *and* K. pneumoniae*. Lane M: 100 bp DNA ladder (Fermentas, USA); Lane 1: negative control (IC); Lane 2: PCR amplicon of* K. pneumoniae*; Lane 3: PCR amplicon of* H. influenzae*; Lane 4: PCR amplicons of multiplex PCR (*K. pneumoniae + H. influenzae)*; Lane 5: PCR amplicons of multiplex PCR (IC +* K. pneumoniae + H. influenzae*).

The multiplex PCR was optimized and standardized with the reference strains of* H. influenzae *and* K. pneumoniae*. As shown in Figure 1, the multiplex PCR assay successfully amplified* p6 *gene of* H. influenzae *(582 bp)*, php *gene of* K. pneumoniae *(202 bp), and* glmM *gene of* H. pylori *(105 bp). The PCR assay was positive for* K. pneumoniae *when* php *gene (202 bp) and IC (105 bp) were present. Meanwhile, the PCR assay was positive for* H. influenzae *when* p6 *gene (582 bp) and IC (105 bp) were detected. The multiplex PCR was considered as valid negative when only IC (105 bp) was observed.

### 3.2. Optimization of Thermostabilized Multiplex Mixture (Enzyme Stabilizer and* Taq* DNA Polymerase)

**Figure 2:**
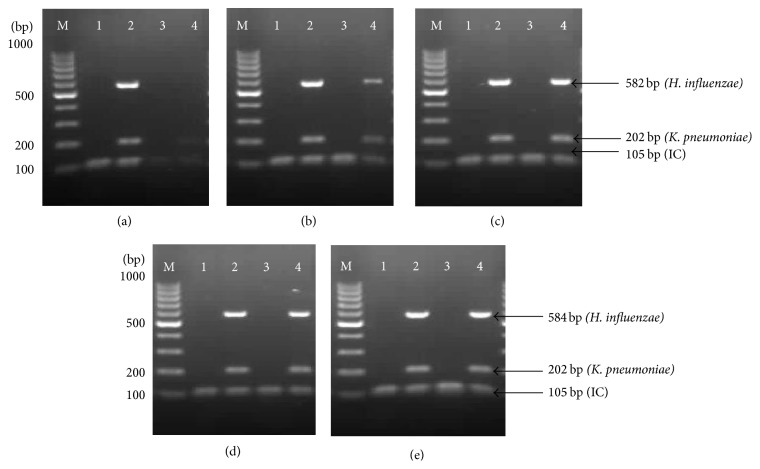
Effect of different concentrations of trehalose on the performance of thermostabilized multiplex PCR. (a) 2% stabilizer. (b) 4% stabilizer. (c) 6% stabilizer. (d) 8% stabilizer. (e) 10% stabilizer. Lane M: 100 bp DNA ladder (Fermentas, USA); Lane 1: negative control before lyophilization (IC); Lane 2: positive control before lyophilization (IC +* K. pneumoniae *+* H. influenzae*); Lane 3: negative control after lyophilization (IC); Lane 4: positive control after lyophilization (IC +* K. pneumoniae *+* H. influenzae*).

**Figure 3:**
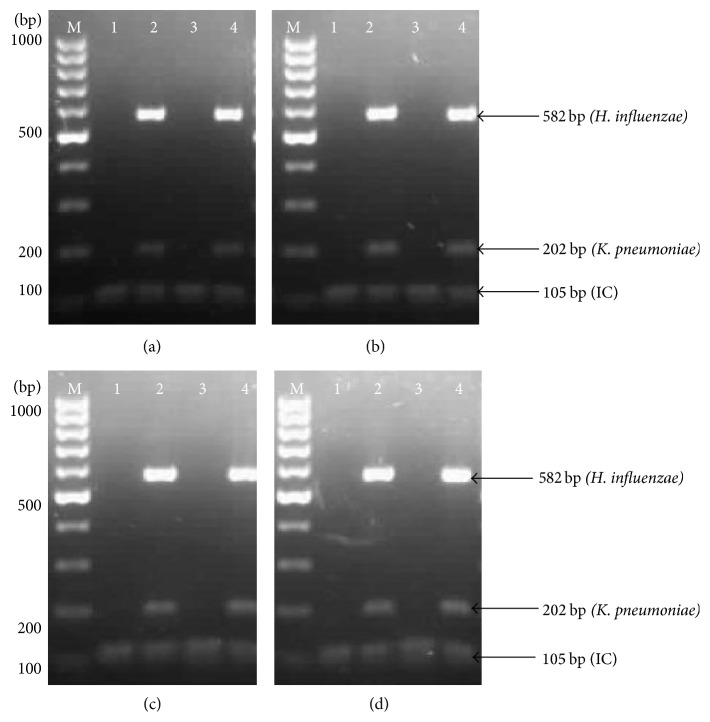
Effect of different concentrations of* Taq *DNA polymerase on the performance of thermostabilized multiplex PCR. (a) 100%* Taq *DNA polymerase. (b) 150%* Taq *DNA polymerase. (c) 200%* Taq *DNA polymerase. (d) 250%* Taq *DNA polymerase. Lane M: 100 bp DNA ladder (Fermentas, USA); Lane 1: negative control before lyophilization (IC); Lane 2: positive control before lyophilization (IC +* K. pneumoniae *+* H. influenzae*); Lane 3: negative control after lyophilization (IC); Lane 4: positive control after lyophilization (IC +* K. pneumoniae *+* H. influenzae*).

Both concentrations of enzyme stabilizer and* Taq *DNA polymerase should be in balance in order to make* Taq *DNA polymerase work at optimum phase. As shown in Figures 2 and 3, 8% of trehalose with 200% (1.5 U) of* Taq *DNA polymerase produced similar band intensities before and after lyophilization process. Therefore, this mixture was selected as the optimal concentration for thermostabilization process.

### 3.3. Evaluation of Analytical Sensitivity of Thermostabilized PCR

**Figure 4:**
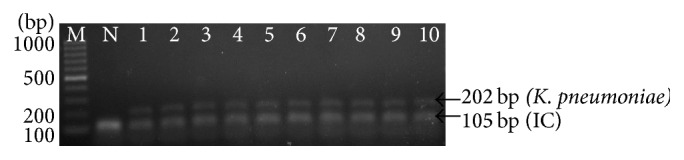
Representative agarose gel electrophoresis of thermostabilized multiplex PCR of* K. pneumoniae *strains. Lane: M, 100 bp DNA ladder (Fermentas, USA); N, negative control; 1,* K. pneumoniae *(ATCC 1706); 2–10, clinical isolates of* K. pneumoniae.*

**Figure 5:**
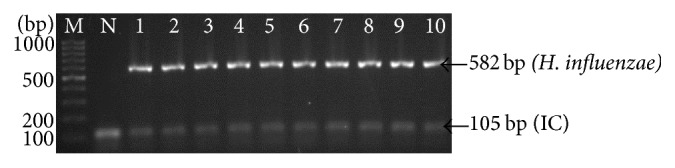
Representative agarose gel electrophoresis of thermostabilized multiplex PCR of* H. influenzae *strains. Lane: M, 100 bp DNA ladder (Fermentas, USA); N: negative control, 1:* H. influenzae *(ATCC 49247); 2–10, clinical isolate of* H. influenzae.*

**Figure 6:**
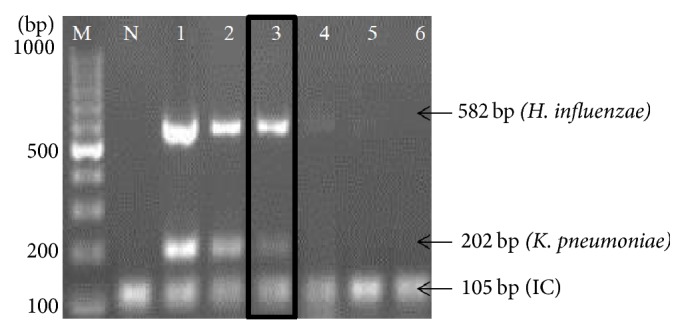
Analytical sensitivity of thermostabilized multiplex PCR at bacterial level for* H. influenzae *and* K. pneumoniae.* Lane: M, 100 bp DNA ladder (Fermentas, USA); N, negative control (IC); 1, positive control (IC +* K. pneumoniae *+* H. influenzae*); 2, 10^5^ CFU/ml lysate of* H. influenzae *and* K. pneumoniae*; 3, 10^4^ CFU/ml lysate of* H. influenzae *and* K. pneumoniae*; 4, 10^3^ CFU/ml lysate of* H. influenzae *and* K. pneumoniae; *5, 10^2^ CFU/ml lysate of* H. influenzae *and* K. pneumoniae; *6, 10^1^ CFU/ml lysate of* H. influenzae *and* K. pneumoniae*.

To evaluate analytical sensitivity of the thermostabilized PCR, we tested the assay with clinical isolates of* K. pneumoniae *(*n* = 20) and* H. influenzae *(*n* = 20) which was previously confirmed by VITEK 2 system (BioMérieux, France). As shown in Figures 4 and 5, the PCR assay successfully amplified all strains of* K. pneumoniae *and* H. influenzae*, respectively. The detection limit of the assay was determined by using 10-fold serial dilutions starting from 10^5^ CFU/ml to 10^1^ CFU/ml. As shown in Figure 6, the detection limit of multiplex PCR was 10^4^ CFU/ml.

### 3.4. Evaluation of Analytical Specificity of Thermostabilized PCR

To verify the analytical specificity of thermostabilized PCR assay, clinical isolates from various bacterial species were tested. The thermostabilized multiplex PCR successfully amplified* H. influenza*e (582 bp),* K. pneumoniae *(202 bp), and IC (105 bp). All other bacterial species were negative, and this demonstrates that no cross-reactivity occurred (Table 1). The analytical specificity of the thermostabilized, one-step, PCR assay was 100%.

### 3.5. Evaluation of Stability of Thermostabilized PCR

**Figure 7:**
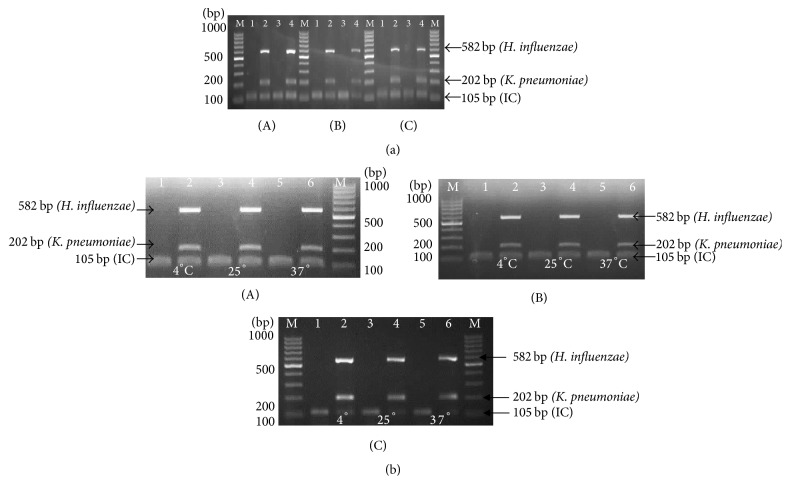
(a) Accelerated stability evaluation at Day 0 storage of test at different temperatures. (A) 4°C. (B) 25°C. (C) 37°C. Lane M: 100 bp DNA ladder (Fermentas, USA); Lane 1: negative control (before thermostabilization); Lane 2: positive control (before thermostabilization); Lane 3: negative control (after thermostabilization); Lane 4: positive control (after thermostabilization). (b) Accelerated stability evaluation of test at different temperatures and storage periods. (A) Day 7. (B) Day 14. (C) Day 30. Lane M: 100 bp DNA ladder (Fermentas, USA); Lane 1: negative control at 4°C; Lane 2: positive control at 4°C; Lane 3: negative control at 25°C; Lane 4: positive control at 25°C; Lane 5: negative control at 37°C; Lane 6: positive control at 37°C.

Evaluation of thermostabilized multiplex PCR stability was determined by time and through accelerated aging test using heat. As shown in Figures 7(a) and 7(b), the thermostabilized was shown to be stable up to 1 month at all temperature values. Based on the band intensities, accelerated stability aging test was used to calculate the shelf life at temperature 37°C. Accelerated stability calculation was performed using estimated shelf life based on conservative standard accelerated aging factor. At elevated temperature of 37°C, the thermostabilized multiplex PCR mixture was stable up to 30 days, indicating the following.

The longest duration that the thermostabilized PCR maintained its activity was calculated as described by Clark, 1991 [9]:

  Age of the thermostabilized PCR tubes = 1.0 months at 37°C.
  Ambient temperature RT = 25°C.
 
*Q*^10^ = 1.8.
  Acceleration factor at 37°C (based on 12°C temperature difference): (1.8)^1.2^ = 2.02.
  Length of time at elevated temperature: 1.0 month.
  Estimation of shelf life:
 
accelerated  age = age × acceleration  factor (at 37°C),
 
1.0  months × 2.02 = 2.02 months,
 
shelf  life = accelerated  age + actual  age,
 
2.02 + 1.0 = 3.02 months.

## 4. Discussion

Conventional culture method of bacterial identification becomes the gold standard in laboratories. However, this conventional method is time consuming, laborious, and less sensitive [4, 5]. There are a lot of factors that may influence the culture results, including antimicrobial treatment, prolonged transportation time, and inappropriate transportation medium [5, 6]. Due to these limitations, the need to utilize more sensitive methods such as multiplex PCR, real-time PCR, and microarray assays are now greatly increased. These approaches provide faster speed and greater sensitivity in the identification of bacteria, especially respiratory pathogens. Detection of* H. influenzae *and* K. pneumoniae *by real-time PCR and microarray has been developed to increase the chances of detection rates and the accuracy of quantification [7–9]. However, there are some challenges that limit the widespread application of PCR such as the lack of cold-chain-free PCR assay, well-trained staffs to reduce the risk of contamination, instrument availability, and good facilities to store all required reagents and enzyme in a specific temperature range [10]. Lyophilization of PCR assays can overcome some of these limitations and enable widespread application of PCR in low resource setting. This study describes the development of a lyophilized, cold-chain-free PCR assay for detection of* H. influenzae *and* K. pneumoniae *in single tube reaction.

In our study, the primers were designed to amplify* php *gene of* K. pneumoniae *and* p6 *gene of* H. influenzae*.* Php *gene was chosen due to its ability to differentiate* K. pneumoniae *and* K. varicella*. Meanwhile,* p6*, the gene that encodes the outer membrane protein, is present in all strains of* H. influenzae *and is able to detect both typeable and nontypeable* H. influenza*. An IC primer was designed based on the* glmM *gene of* H. pylori, *which is genetically unrelated to both* K. pneumoniae *and* H. influenzae. *The IC was incorporated in order to ensure the result's validity and to identify PCR inhibitory factors [11]. Lyophilization of PCR assay has many advantages and is important in mitigating some of the PCR limitations. In the present study, the developed multiplex PCR assay was then lyophilized into a ready-to-use format which contains all reagents for PCR reactions, including primers and* Taq *DNA polymerase, requiring only the addition of water and extracted sample for analysis. Two parameters (enzyme stabilizer and* Taq *DNA polymerase) were optimized during this process. Trehalose was added as an enzyme stabilizer to protect and prevent any conformation changes of* Taq *DNA polymerase enzyme during and after dehydration [11, 12]. It stabilizes the PCR reagents, especially* Taq *DNA polymerase against stresses brought by vacuum drying and freeze thawing [13]. The presence of glycerol may affect the product's stability. The improved sensitivity observed in lyophilized assay can be explained by the fact that when developing these assays, the* Taq *DNA polymerase used is glycerol-free. This allows higher concentrations of* Taq *DNA polymerase to be used per reaction in the lyophilized assay than in the nonlyophilized assay [14]. The intensities of bands were found to be increasing from 2 to 10% stabilizer. Hence, enzyme stabilizer at the concentration of 8% was chosen in this study since it gives almost the same band intensities before and after lyophilization. All bands showed uniform amplicon intensities at* Taq *DNA concentration of 200%. Lower concentration of* Taq *DNA polymerase gave less band intensities and may affect the efficacy and specificity of amplification process [11]. Based on the results obtained, the optimum conditions of thermostabilized PCR were determined at 8% enzyme stabilizer and 200%* Taq *DNA polymerase.

The sensitivity and specificity performances of thermostabilized multiplex PCR were evaluated using 127 clinical isolates from various bacteria strains. Among 120 isolates, 20 isolates were identified as* H. influenzae *and 20 others were identified as* K. pneumoniae. *The specificity of the assay was assessed by testing a panel of other bacterial species which were previously identified using VITEK 2 system (BioMérieux, France) (Table 1). All* non-H. influenzae *and* non-K. pneumoniae* showed negative results where only IC amplicon was detected. An overall analysis of the multiplex PCR showed 100% sensitivity and specificity. The detection limit of thermostabilized multiplex PCR for* H. influenzae *and* K. pneumoniae *at bacteria level was at 1.0 × 10^3^ CFU/ml, whereas the sensitivity of both multiplex PCR assays was at 1.0 × 10^4^ CFU/ml. The sensitivity level achieved in this study was found to be comparable to the ones obtained in prior studies [3, 11, 12].

Using the* Q*^10^ method, the stability of the PCR assay was estimated using accelerated aging techniques at elevated temperature and monitoring stability overtime. The longest duration that the thermostabilized PCR maintains its activity is calculated to determine the shelf life of the assay [14]. The stability of thermostabilized PCR assay was tested by comparing the performance of assay at three different temperatures (4°C, 25°C, and 37°C) over a period of 30 days (estimated 1 month), tested at seven-day intervals. Based on the results, thermostabilized multiplex PCR was stable at 37°C of elevated temperature for 30 days and, from calculation, 3.02 months (conservative calculation) when kept at 37°C. The stability of PCR assay demonstrated that the assay can be transported and stored without the need for cold-chain and being concerned towards the degradation of reagents or low test reliability.

## 5. Conclusion

The thermostabilized, ready-to-use, cold-chain-free, multiplex PCR was successfully developed for simple, rapid, and simultaneous detection of* H. influenzae *and* K. pneumoniae. *This method requires only two pipetting steps and has simple workflow and very low risk of contamination and is stable at room temperature.

## Figures and Tables

**Table 1:** Bacterial species and strains used in this study and results of the thermostabilized mPCR.

No	Reference strains	Php (202 bp)	P6 (582 bp)	Internal control (IC)
(1)	*Klebsiella pneumoniae *(ATCC 1706)^a^	+	−	+
(2)	*Klebsiella pneumoniae *(*n* = 20)^b^	+	−	+
(3)	*Haemophilus influenzae *(ATCC 49247)^a^	−	+	+
(4)	*Haemophilus influenzae *(*n* = 20)^b^	−	+	+
*Gram negative bacteria*				
(5)	*Pseudomonas aeruginosa *(*n* = 15)^b^	−	−	+
(6)	*Escherichia coli (EHEC) *(*n* = 6)^b^	−	−	+
(7)	*E. coli (ETEC) *(*n* = 1)^b^	−	−	+
(8)	*Shigella sonnei *(*n* = 1)^b^	−	−	+
(9)	*Shigella flexneri *(*n* = 1)^b^	−	−	+
(10)	*Shigella boydii *(*n* = 1)^b^	−	−	+
(11)	*Shigella dysenteriae *(*n* = 1)^b^	−	−	+
(12)	*Salmonella typhi *(*n* = 1)^b^	−	−	+
(13)	*Vibrio cholerae *(*n* = 1)^b^	−	−	+
(14)	*Citrobacter freundii *(*n* = 1)^b^	−	−	+
(15)	*Acinetobacter baumannii *(*n* = 1)^b^	−	−	+
(16)	*Acinetobacter *spp.(*n* = 1)^b^	−	−	+
(17)	*Klebsiella *spp.(*n* = 3)^b^	−	−	+
(18)	*Haemophilus parainfluenzae *(*n* = 2)^b^	−	−	+
(19)	*Proteus mirabilis *(*n* = 1)^b^	−	−	+
(20)	*Burkholderia pseudomallei *(*n* = 1)^b^	−	−	+
(21)	*Moraxella catarrhalis *(*n* = 1)^b^	−	−	+
(22)	*Citrobacter *spp.(*n* = 1)^b^	−	−	+
(23)	*Mycobacterium tuberculosis *(*n* = 6)^b^	−	−	+
(24)	*Mycobacterium bovis *(*n* = 1)^b^	−	−	+
*Gram positive bacteria*				
(25)	*Listeria monocytogenes *(*n* = 1)^b^	−	−	+
(26)	*Staphylococcus aureus *(*n* = 25)^b^	−	−	+
(27)	Methicillin Resistant *Staphylococcus aureus *(MRSA) (*n* = 7)^b^	−	−	+
(28)	*Streptococcus viridians *(*n* = 1)^b^	−	−	+
(29)	*Streptococcus pneumoniae *(*n* = 1)^b^	−	−	+
(30)	*Streptococcus pyogenes *(*n* = 1)^b^	−	−	+
(31)	*Streptococcus *Group A (*n* = 1)^b^	−	−	+
(32)	*Streptococcus *Group B (*n* = 1)^b^	−	−	+
(33)	*Streptococcus *Group G (*n* = 1)^b^	−	−	+
(34)	*Bacillus subtilis *(*n* = 1)^b^	−	−	+

^a^Reference strains from America Type Control Center (ATCC), USA.

^b^Department of Medical Microbiology and Parasitology, School of Medical Sciences, Universiti Sains Malaysia.
